# Selection signature analysis reveals genes associated with tail phenotype in sheep

**DOI:** 10.3389/fgene.2024.1509177

**Published:** 2024-12-11

**Authors:** Yunxia Qi, Xiaolong He, Biao Wang, Chaoyun Yang, Lai Da, Bin Liu, Wenguang Zhang, Shaoyin Fu, Yongbin Liu

**Affiliations:** ^1^ College of Animal Sciences, Xichang University, Xichang, China; ^2^ Animal Husbandry Institute, Inner Mongolia Academy of Agricultural and Animal Husbandry Sciences, Hohhot, China; ^3^ Inner Mongolia BIONEW Technology Co., LTD., Hohhot, China; ^4^ College of Life Sciences, Inner Mongolia Agricultural University, Hohhot, China; ^5^ State Key Laboratory of Reproductive Regulation and Breeding of Grassland Livestock (R2BGL), College of Life Sciences, Inner Mongolia University, Hohhot, China

**Keywords:** sheep, tail type, selection signature analysis, F_ST_, π-ratio, XP-EHH

## Abstract

Tail type of sheep, which may be affected by many genes with a complex mechanism, is an important economic trait concerned by both raiser and consumers. Here, we employed two sheep breeds with extreme phenotypes - Mongolian sheep (short-fat-tailed) and Bamei Mutton sheep (long-thin-tailed) to analyze the genetic differences at the genomic level and find candidate genes associated with tail phenotype. The results of population structure analysis showed that the LD decay rate of Mongolian sheep was greater than that of Bamai Mutton sheep. When K = 2, the two populations were obviously separated with a certain degree of mixing. From 49 sheep individuals, 20,270,930 and 2,479,474 SNPs and Indels were identified, respectively. Selection signals were detected based on F_*ST*_, π-Ratio, and XP-EHH. These three methods identified 85 candidate genes, of which *PDGFD, GLIS1, AR,* and *FGF9* were reported to be associated with tail fat deposition, while *VRTN* associated with tail length in sheep tail phenotype; the others were novel genes that may play important roles in sheep tail phenotype formation. Gene annotation revealed that these candidate genes mainly participate in pathways associated with fat deposition or lipid metabolism. This study provided insight into sheep tail type development and a guide for molecular breeding.

## 1 Introduction

Sheep (*Ovis aries*), as a major source of meat, milk, fiber and leather for mankind, has been domesticated since Mesolithic period, ∼11,000 years ago (Chessa et al., 2009; Alberto et al., 2018; Deng et al., 2020). During this long procedure sheep has evolved diverse phenotypes such as coat color, horn, tail type, etc., under natural and artificial selection (Kijas et al., 2009). Of these phenotypes, tail type is an important economic trait concerned by both producers and consumers. According to its length and fat deposition, sheep tail can be divided into five major types: long-fat tail, short-fat tail, long-thin tail, short-thin tail, and fat-rumped tail. It is widely believed that the wild ancestors of sheep were thin-tailed, while the fat-tailed sheep breeds emerged as an adaptive response to harsh and challenging environmental conditions (such as climate fluctuation, drought, and food scarcity) (Atti et al., 2004; Pourlis, 2011; Moradi et al., 2012; Kalds et al., 2021). Fat-tailed sheep could deposit up to 20% of their carcass weight as fat in the tail (Yousefi et al., 2012). The large amount of tail fat serves as an energy source for sheep, and also provided people valuable edible fat in the era of material scarcity. Nowadays, however, with the increasing incidences of obesity and cardiovascular disease, people prefer a diet low in fat and high in protein. On the other hand, with the popularization of intensive and semi-intensive feeding management, fat-tail of sheep has lost its original advantages and brought inconvenience to production management, such as inconvenience for mating (Kridli and Said, 1999) and locomotion (Orihuela and Ungerfeld, 2019). In addition, a large amount of fat deposition in tails may reduce feed conversion rate and even affect the carcass quality (Safdarian et al., 2008; Yousefi et al., 2012). Yousefi *et al.* found that the thin-tailed breed accumulated more intramuscular fat in *longissimus dorsi* muscle and had lower shear force and better eating quality, tenderness, and drip loss than the fat-tailed breed (Yousefi et al., 2012). In practice, tail docking (O’Donovan et al., 1973; Shelton et al., 1991; Bicer et al., 1992; Moharrery, 2007; Wang et al., 2018) and cross-breeding (Kashan et al., 2005; Khaldari et al., 2008; Abdullah et al., 2010) are usually taken to reduce tail size and length. It was reported that tail docking may improve lambs’ growth, slaughter performance and mutton quality (Atti and Mahouachi, 2011; Marai et al., 1987; Bicer et al., 1992; Abouheif et al., 1993; Bing et al., 2006). However, tail docking is stressful and risky, and has been banned in several countries to improve animal welfare (Eck et al., 2019). Cross-breeding takes time and efforts, and the results are usually unsatisfactory. Currently, how to breed short-thin-tailed sheep through molecular breeding methods has become a focus of sheep breeders, and the key to solve this problem is to identify genes related to tail phenotype of sheep. There were some research on tail phenotype and several promising genes such as *PDGFD*, *BMP2* and *TBXT*, etc., associated with tail phenotype had been suggested (Yuan et al., 2017; Zhi et al., 2018; Dong et al., 2020; Mastrangelo et al., 2019; Pan et al., 2019; Moradi et al., 2012), but most of the studies focused either on fat deposition or tail length, and the results are usually inconsistent. The genetic mechanics underlying tail phenotype still remain unclear.

**FIGURE 1:**
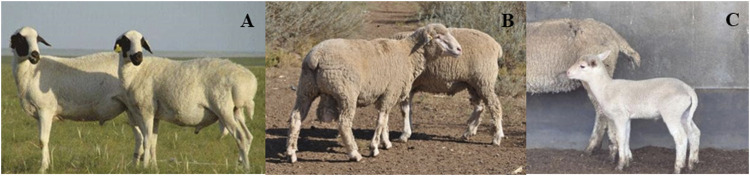
Tail phenotype of sheep. **(A)** Short-fat-tailed Mongolian sheep. **(B)** Long-thin-tailed Bamei Mutton sheep after docking. **(C)** long-thin-tailed lamb of Bamei Mutton sheep before docking.

Mongolian sheep, short-fat-tailed (Figure 1A), is the most widely distributed and abundant sheep breed in China. It is mainly distributed in Inner Mongolia Autonomous Region, northeast, north, and northwest of China. Mongolian sheep is an ancient indigenous breed formed by natural and artificial selection for a long time, and is favored by local herdsman and consumers because of its rough feeding resistance, cold resistance, drought resistance, and high-quality meat. Bamei Mutton sheep, which is long-thin-tailed (Figures 1B, C), is the first dual-purpose breed that was bred in China by crossing local fine-mixed sheep as the maternal line with German Merino sheep as the paternal line. It contains 6.25% bloodline of Mongolian sheep. Bamei Mutton sheep is mainly distributed in Bayannur City of Inner Mongolia Autonomous Region, China. It is characterized by resistance to rough forage, strong stress resistance, good adaptability, rapid weight gain in lamb fattening, and early sexual maturity. In the present study, we performed whole genome resequencing of the two breeds with extreme tail phenotypes to investigate selection signatures and candidate genes associated with tail phenotype (fat vs. thin and long vs. short) based on three statistical tests, including fixation index (F_*ST*_), π-Ratio, and cross-population extended haplotype homozygosity test (XP-EHH). The candidate genes identified in our study provided the basis for understanding the molecular mechanism of tail phenotype in sheep.

## 2 Materials and methods

### 2.1 Sample collection, DNA extraction, and sequencing

A total of 28 Mongolian sheep (MG) and 21 Bamei Mutton sheep (BM) were selected from Inner Mongolia Autonomous Region, China. The sheep were raised and managed under the same condition. All individuals were typical of the breeds and unrelated according to pedigree records or owner’s information. Blood samples were collected and returned to laboratory on dry ice. Genomic DNA was extracted from the blood samples following the standard phenol-chloroform extraction procedure. DNA samples that passed the test (D260 nm/D280 nm = 1.7–1.9) were randomly interrupted into fragments of 500 bp in length. Paired-end sequencing libraries were constructed according to the manufacturer’s instructions (Illumina Inc., San Diego, CA, USA) and sequenced on the Illumina HiSeq Xten Sequencer (Illumina Inc.) with PE150 module.

### 2.2 Alignments and variant identification

After filtering out low quality reads, the 150-bp paired-end clean reads were mapped onto the sheep reference genome Oar v.4.0 (https://www.ncbi.nlm.nih.gov/assembly/GCF_000298735.2) with BWA-MEM using the default parameters (Li and Durbin, 2009). After alignments, SNP calling was performed using SAMtools and Genome Analysis Toolkit (GATK, v.3.8) (Nekrutenko and Taylor, 2012). All SNPs were filtered using the ‘Variant Filtration’ module of GATK with the standard parameters as below: Window 4; Variants with QD (quality depth) < 4.0; FS (Phred-scaled *p*-value using Fisher’s exact test to detect strand bias) > 60.0; MQ < 40.0; -G_filter “GQ < 20”.

The implementation of SAMtools mpileup (v.1.8) (Li, 2011) was run in a multi-sample mode to calculate genotype likelihoods from the aligned reads for all samples simultaneously. The parameters *-E* and *-t* were used to recalculate (and apply) base alignment quality and produce per-sample genotype annotations, respectively. Then, the estimated genotype likelihoods were converted into genotypes using *BCFtools* call using the *-v* and *-m* flags to output variable sites only, and permitted sites to have more than two alternative alleles, respectively.

Based on the annotation file of the sheep reference genome Oar v.4.0, a transcript FASTA file for database was built using the retrieve_seq_from_fasta.pl module of ANNOVAR, and then the functional annotation for each SNP was performed using the table_annovar.pl module of ANNOVAR (Wang et al., 2010).

### 2.3 Population structure analysis

SNPs were pruned the in high levels of pair-wise LD using PLINK v.1.9 (Purcell et al., 2007) with the parameter (−-indep-pair-wise 50 5 0.2) to perform principal component analysis (PCA) and ADMIXTURE analysis. PCA of whole-genome SNPs for all 49 individuals was conducted using the GCTA v.1.24.2 (Yang et al., 2011). Furthermore, population structure analysis was carried out using the ADMIXTURE v1.3 (Alexander and Lange, 2011) with kinship (*K*) ranged from 2 to 5. The unrooted Neighbor-joining (NJ) tree was constructed with TASSEL using the matrix of pairwise genetic distances and visualized with iTOL (https://itol.embl.de/). The LD decay for each group was measured using PopLDdecay (Zhang et al., 2019) with default parameters.

### 2.4 Genome-wide selective sweep test

To identify the selective sweep regions, we performed genome-wide scans of selection signals using three metrics: allele frequency based methods F_*ST*_ (Weir and Hill, 2002), π-Ratio (Danecek et al., 2011), and haplotype-based method XP-EHH (Sabeti et al., 2007a).

The SNPs were filtered with parameters (--maf 0.05 -max-missing 0.90) using PLINK v.1.9 (Purcell et al., 2007). The F_*ST*_ was calculated using VCFtools (Danecek et al., 2011) with parameter “--weir-fst-pop group1 --weir-fst-pop group2 --fst-window-size 50000 --fst-window-step 20000 --maf 0.05 --max-missing 0.90”. Then the F_*ST*_ values were normalized (ZF_*ST*_) using the Ztransformation method (Rubin et al., 2010). The genetic diversity (π-Ratio) was calculated using VCFtools with parameters “--keep gropu1/gropu2 --window-pi 50000 --window-pi-step 20000 --maf 0.05 --max-missing 0.90” and python scripts. The overlap of the top 5% windows in each method was considered as candidate signatures of selection.

The XP-EHH was performed for every SNP using the default settings by selscan v.1.1 (Szpiech and Hernandez, 2014), and genotypes were phased using Beagle (Browning and Browning, 2007) with default parameters. The genome-wide raw XP-EHH statistics were standardized to a distribution with zero mean and unit variance. SNPs in the top 0.1% are taken as significant SNPs. Significant regions are identified by combining SNPs of significant XP-EHH scores that are less than 200 kb apart. If two SNPs both have significant XP-EHH scores and were less than 200 kb apart, then the two SNPs formed a region.

In the π-Ratio and XP-EHH tests, the BM sheep were used as the target population, and the MG sheep as the reference population.

### 2.5 Gene ontology enrichment and KEGG pathway analyses

According to genome annotation, a gene was assumed to be under positive selection if it overlapped with a selection signal. To obtain an in-depth view of the biological significance of the candidate genes, online Gene Ontology (GO) enrichment and Kyoto Encyclopedia of Genes and Genomes (KEGG) pathway analyses were conducted by retrieving *O. aries* in self-built database (No.AH96240) on AnnotationHub website (https://annotationhub.bioconductor.org/). Protein-Protein Interaction (PPI) analysis was performed using STRING database (https://cn.string-db.org/).

## 3 Results

### 3.1 Overview of sequencing quality

After sequencing and data quality control, more than 100 million clean reads were obtained in the MG and BM groups, respectively. The number of clean bases in the BM group was found to be more than 4G greater than that in the MG group. The mapping rate is greater than 98% in both groups with an average depth of 7.31 × (Table 1; Supplementary Table S1).

**TABLE 1:** Overview of sequencing statistics.

Group	Clean reads number	Clean bases number (G)	Map rate (%)	Effective depth
MG	113,552,917	17.03	99.30	6.47
BM	143,757,312	21.56	98.73	8.14

### 3.2 SNP identification

**FIGURE 2:**
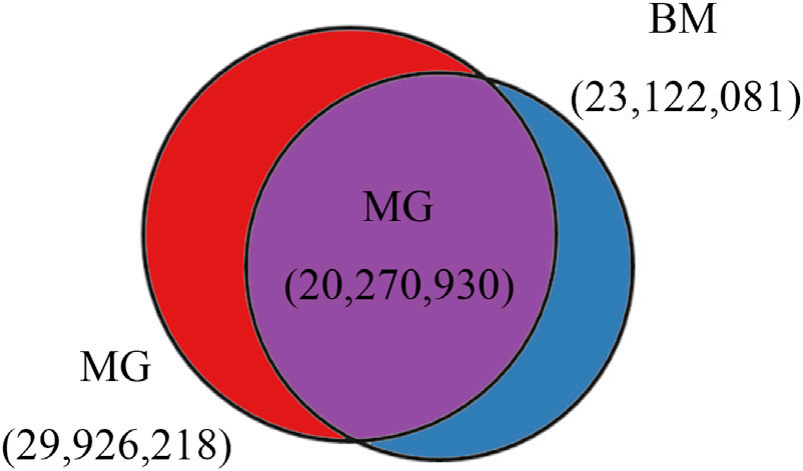
SNPs identified in BM and MG sheep.

In total, 29,926,218 and 23,122,081 SNPs, 3,755,604 and 5,005,409 Indels were identified in MG and BM sheep, respectively, among which, 20,270,930 SNPs and 2,479,474 Indels are common in MG and BM sheep (Figure 2). Functional annotation of the polymorphic sites showed that the vast majority of SNPs and Indels were present either in intergenic regions (62.2% and 60.8%, respectively) or in intronic regions (34.8% and 36.3%, respectively). Exons contained 0.60% of the total variation (Table 2). These results indicate that the variants on the MG and BM genomes differ.

**TABLE 2:** Annotation of SNPs/Indels.

Variant type	SNP count	Indel count
Intergenic	12,609,201	1,782,495
Intronic	7053401	1062533
Exonic	135627	3,549
Downstream	119577	20132
UTR3	118337	23552
Upstream	111348	18491
UTR5	90819	14489
ncRNA intronic	25381	3,850
Upstream or downstream	3,822	655
ncRNA exonic	2,923	282
Splicing	412	266
Exonic, splicing	16	23

### 3.3 Population genetic structure

**FIGURE 3:**
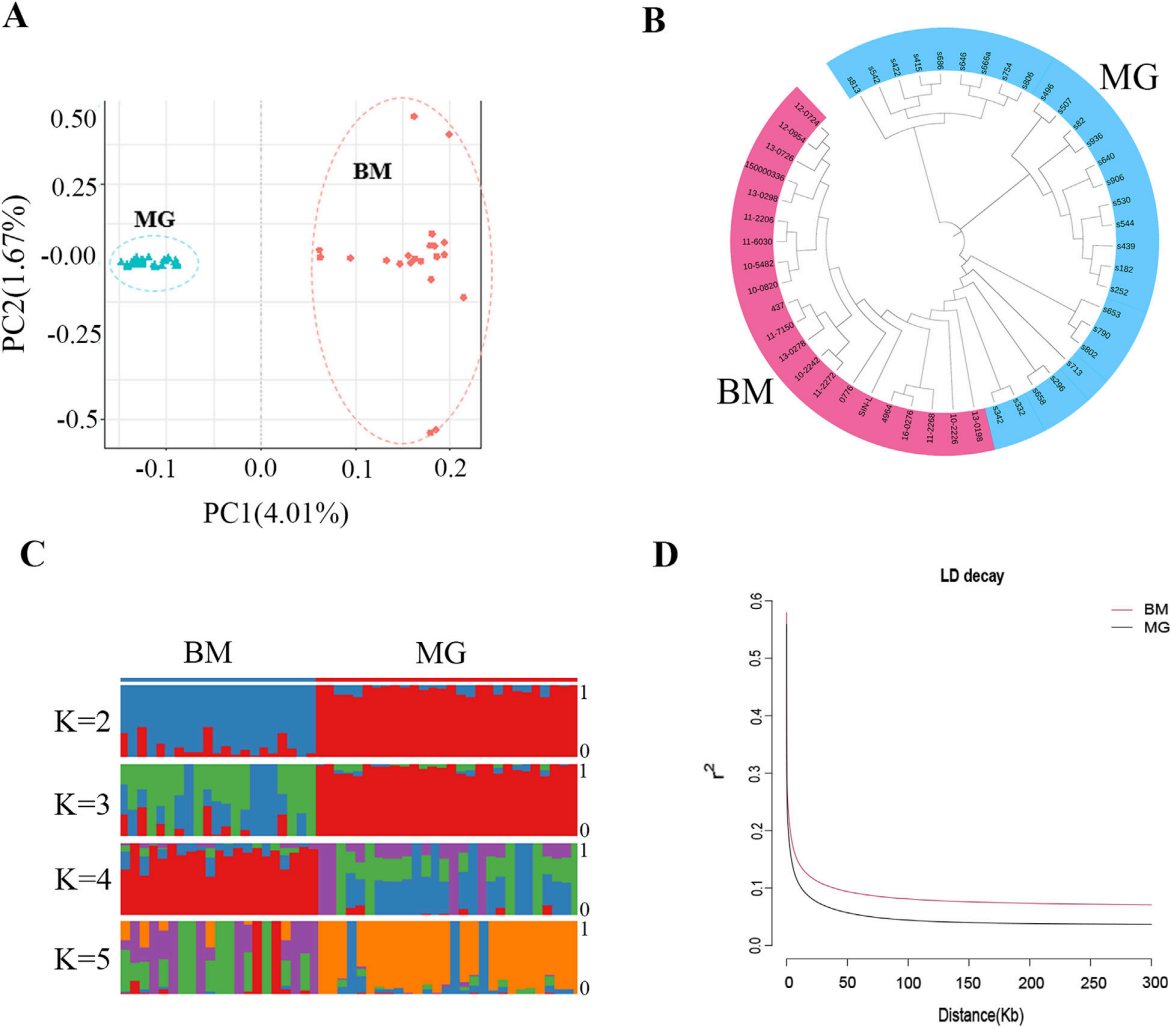
Population genetic structures of BM and MG sheep. **(A)** Principal component analysis (PCA) of 49 sheep individuals. **(B)** Neighbor-joining (NJ) tree constructed from SNPs data of the two populations. **(C)** Model-based clustering of sheep individuals using ADMIXTURE with *K* = 2–5. **(D)** Correlation coefficients (*r* ^2^) were calculated for the MG and BM sheep over 50-kb windows.

Following the identification of the SNPs, PCA, phylogenetic relationship analysis, and population genetic structure analysis were conducted for all the individuals. The PCA results showed that the BM and MG sheep were clearly separated (Figure 3A), and the NJ tree also produced similar results, with BM and MG sheep divided into 2 clades (Figure 3B), which indicated that there is a certain degree of genetic distance between BM and MG sheep. The results of population genetic structure (Figure 3C) showed that for the BM group, the consistency within groups was better when *K* = 2 and *K* = 4, while for the MG, it was better when *K* = 2 and *K* = 3, indicating that the consistency of the individuals within the groups was better, and that differences existed between the groups. Furthermore, when *K* = 2, BM and MG sheep were obviously divided into two subgroups, but with a certain degree of mixing, which was consistent with the fact that BM sheep contains a certain amount of ancestry of MG sheep. When *K* = 3, no new subgroups appeared in the experimental population. The aforementioned results demonstrate that there are distinctions between BM and GM sheep, which may be attributed to tail phenotype variation. In order to further explore the genetic diversity, linkage disequilibrium, in terms of the correlation coefficient (*r* ^*2*^), was calculated for BM and MG sheep populations. As shown in Figure 3D, the faster LD decay was observed in the MG population, which indicates that MG sheep had higher genetic diversity, and BM sheep had higher degree of domestication and greater intensity of selection.

### 3.4 Detection of selective sweeps

**FIGURE 4:**
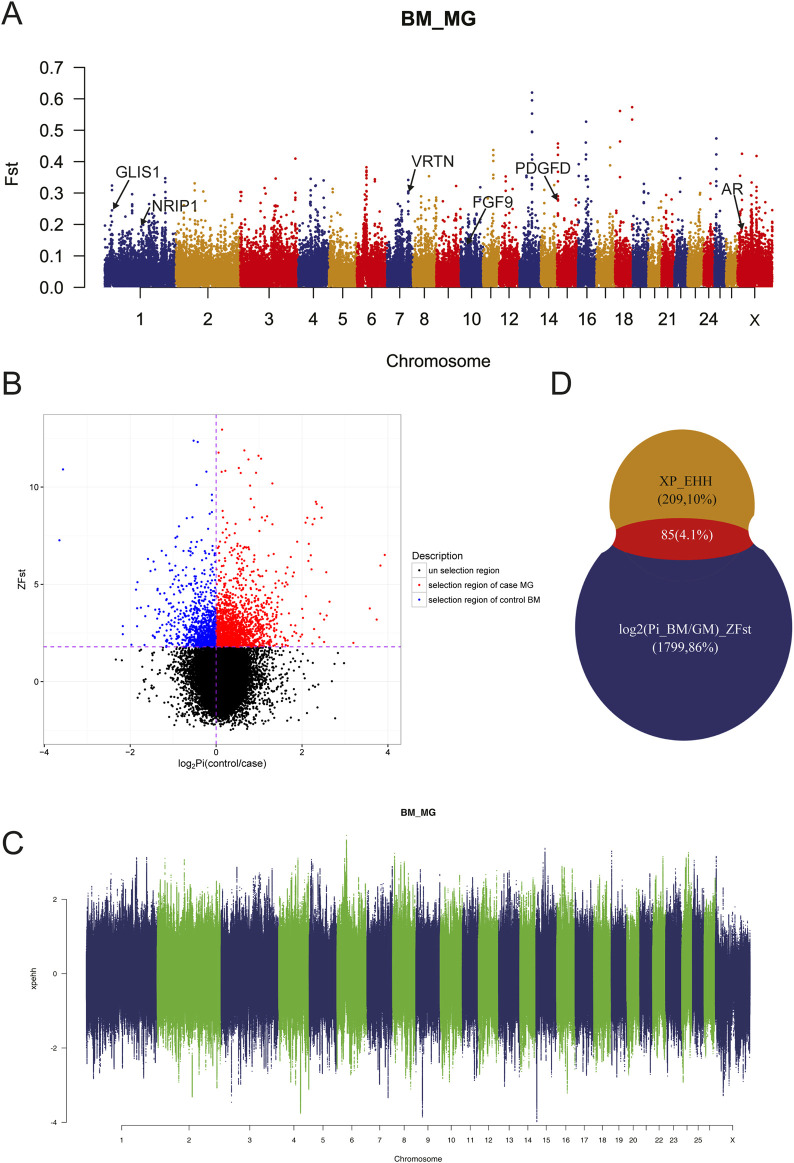
Genomic Selection analyses. **(A)** Selective signals detected by F_*ST.*_ **(B)** Selective signals detected by log2 (Pi_BM/GM)_ZF_*ST.*_ **(C)** Selective signals detected by XP_EHH. **(D)** Overlapping genes identified by log2 (Pi_BM/GM)_ZF_*ST*_ and XP_EHH.

Due to the genetic separation between MG and BM sheep, selective sweep analysis using F_*ST*_, π-Ratio and XP-EHH were performed to investigate selection signals in BM sheep, which would facilitate the identification of target genes. The results demonstrated that the majority of SNPs exhibited moderate genetic variance within the population (Figure 4A, F_*ST*_ < 0.15). Additionally, some SNPs exhibited high genetic variance and high genetic variability on chromosomes 13, 16, and 17, suggesting that these SNPs may be mutations specific to the BM and MG populations. A further 544,123 SNPs were identified under the conditions of F_*ST*_ ≥ 0.2 and π-Ratio ≤0.397 (Figure 4B). The combined analysis of F_*ST*_ and π-Ratio revealed that 1884 genes (representing the top 10% of genes) were identified by log2 (Pi_BM/GM)_ZF_*ST*_, while 294 genes were identified by XP_EHH (Figure 4C). Eventually, a total of 85 overlapping genes were identified as candidate genes by log2 (Pi_BM/GM)_ZF_*ST*_ and XP_EHH (Figure 4D; Supplementary Table S2). Of these candidate genes, some were known to be related to sheep tail phenotype, such as fat deposition associated genes *PDGFD*, *GLIS1*, *AR, FGF9,* and vertebral number variation associated gene *VRTN*; some were novel genes that may have relationship with sheep tail phenotype formation.

### 3.5 Function annotation of the selected genes

**FIGURE 5:**
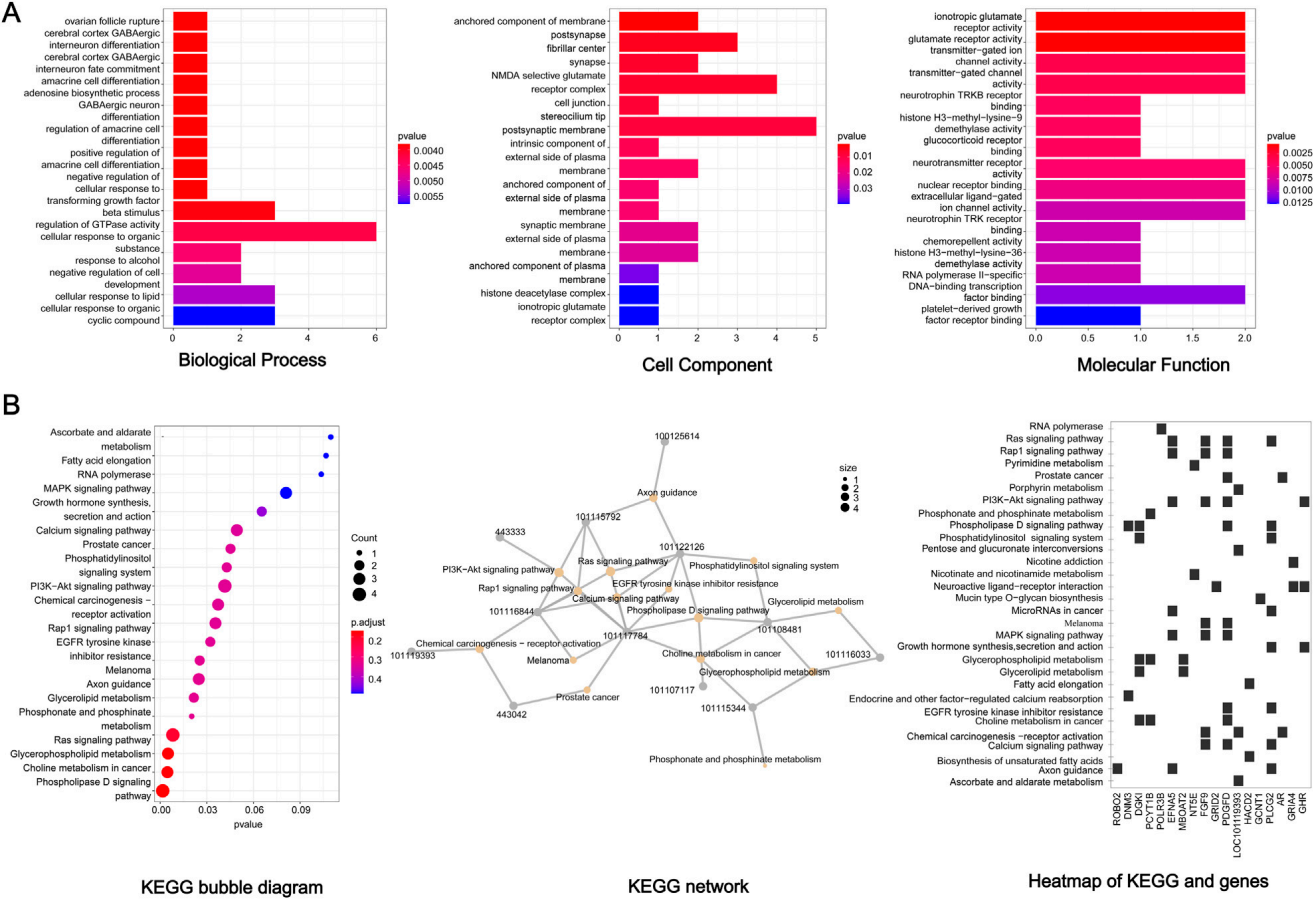
GO and KEGG analysis of the 85 candidate genes. **(A)** GO analysis. **(B)** KEGG analysis.

**FIGURE 6:**
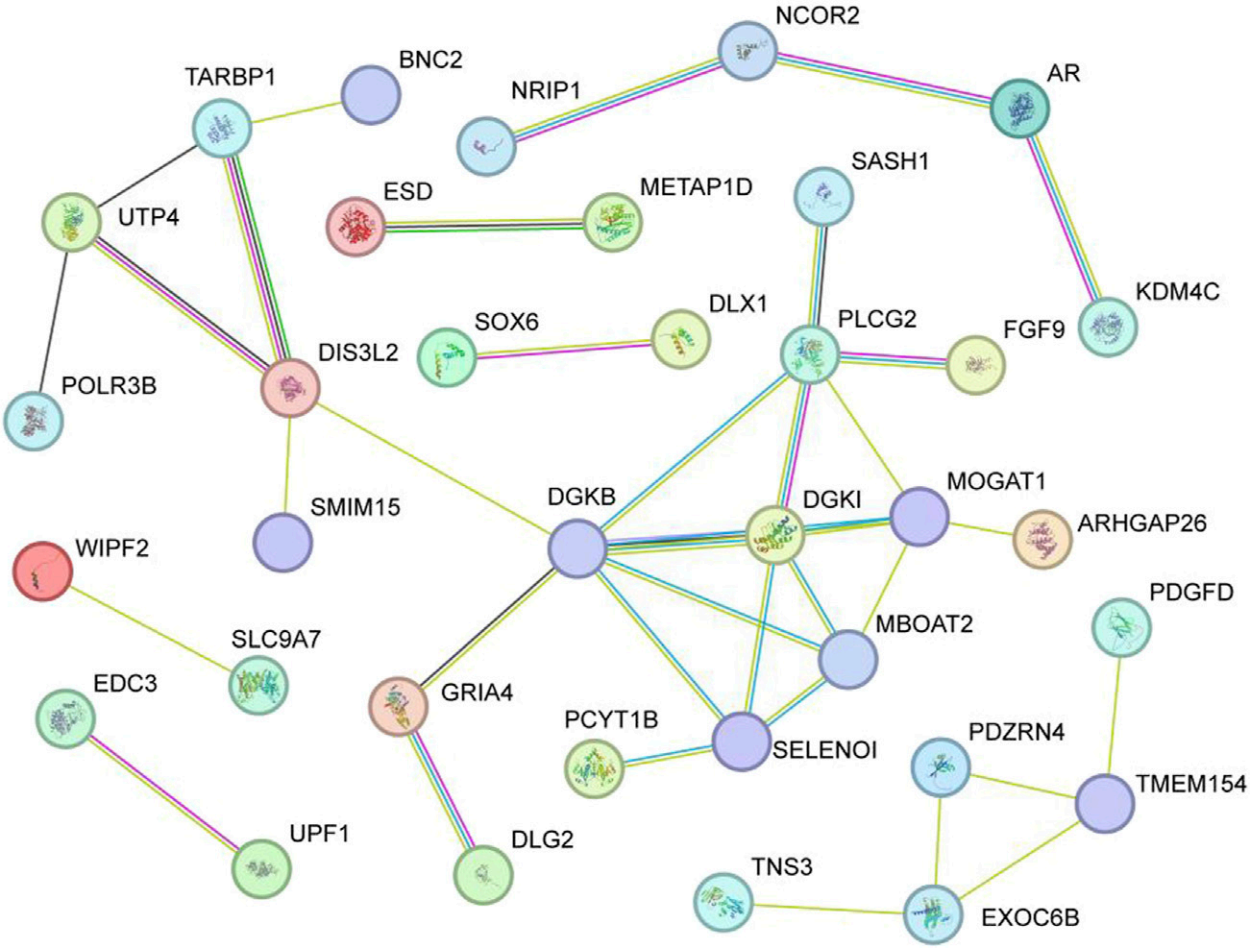
Protein-Protein Interaction (PPI) of the 85 candidate genes.

Functional association of the 85 candidate genes was further investigated by GO and KEGG analysis. For GO analysis, the biological processes mainly focused on ‘cellular response to organic substance’, ‘cellular response to lipid’, ‘cellular response to organic cyclic compound’, ‘negative regulation of cell development’, ‘beta stimulus regulation of DTPase activity’, and so on (Figure 5A) (*p* < 0.05). In terms of KEGG, several signaling pathways related to lipid metabolism were significantly enriched, including ‘phospholipase D signaling pathway’, ‘glycerophospholipid metabolism’, ‘glycerolipid metabolism’, and ‘fatty acid elongation’ (Figure 5B). In addition, some significant pathways were also enriched, such as ‘MAPK pathway’, ‘P13K Akt signaling pathway’, ‘Calcium signaling pathway’ and ‘ras signaling pathway’. The interaction of KEGG pathways and the relationship between genes and pathways was show in Figure 5B. Furthermore, these candidate genes showed close functional association (Figure 6).

## 4 Discussion

In the past decade, the improvement of sheep tail phenotype has gradually developed from utilization of traditional hybridization or tail-docking to molecular breeding technology. To date, several methods are employed to detect the selective sweeps in various livestock genomes. In the present study, we used three metrics, allele frequency-based methods F_*ST*_, π-Ratio, and haplotype-based method XP-EHH, to identify genome-wide selective sweep regions. The power of each test was different, and any set of candidate genes may contain some false positives (Nielsen et al., 2007). F_*ST*_ measures the genetic differentiation between populations (Holsinger and Weir, 2009); π-Ratio identifies the differences in nucleotide divergence between populations (Sun et al., 2020); XP-EHH detects ongoing or nearly fixed selective sweeps by comparing haplotypes between the two populations (Sabeti et al., 2007b). Combining multiple tests can improve the power of detecting selection signatures (Zeng et al., 2007), making the results more reliable. We considered the overlapping genes derived from three methods as candidate genes and eventually identified 85 candidate genes. Among these genes, *PDGFD* (Dong et al., 2020; Zhu et al., 2021; Pan et al., 2019; Mastrangelo et al., 2019; Zhao et al., 2020; Li et al., 2020a; li et al., 2020b; Wang et al., 2022; Wei et al., 2015; Luo et al., 2021), *GLIS1* (Luo et al., 2021), *NRIP1* (*RIP140*) (Xu et al., 2017), *AR* (Moradi et al., 2022), *FGF9* (Moioli et al., 2015), and *VRTN* (Mastrangelo et al., 2019; Zhu et al., 2021; Moioli et al., 2015) were formerly reported to be involved in regulation of tail fat deposition or tail length.

Many studies have recently highlighted the *platelet-derived growth factor D* (*PDGFD*) gene as a new sheep tail phenotype pattern maker (Wei et al., 2015; Pan et al., 2019; Dong et al., 2020; Li Q. et al., 2020; Li X. et al., 2020; Luo et al., 2021; Mastrangelo et al., 2019). Dong *et al.* found that the expression of the *PDGFD* gene is higher in fat-tailed breeds than in thin-tailed breeds, and a similar result was observed in obese mice and human after analyzing a public transcriptomic data (Dong et al., 2020). Overexpression of *PDGFD* in ovine preadipocytes could promote adipogenic differentiation, and the expression levels of two adipogenesis marker genes (*PPARc* and *LPL*) increased after *PDGFD* overexpression (Li Q. et al., 2020). Furthermore, oil red O staining showed that the number of lipid drops was higher in the PDGFD-overexpressing group than in the control group (Li Q. et al., 2020). These studies indicated that *PDGFD* gene plays a positive regulation role in the fat deposition process of sheep tail. Interestingly, there were also different discoveries about the expression profile of the *PDGFD* gene. Li X. et al. (2020) identified four *PDGFD* transcripts (I, II, III, and IV), and transcript I was the most differentially expressed transcripts between the thin-tailed and the fat-tailed/fat-rumped sheep breeds. Notably, *PDGFD* expression (at the mRNA and protein levels) was consistently negatively correlated with fat deposition in sheep tails (Li X. et al., 2020). The highest *PDGFD* gene expression level was observed in the thin-tailed Chinese Merino sheep, followed sequentially by the small fat-tailed Han sheep, the large fat-tailed Han sheep, and the fat-rumped Altay sheep (Li X. et al., 2020). The authors inclined to the idea of involving the PDGFRb signaling (a receptor of PDGFD) in inhibiting the differentiation of white adipocytes by regulating the expression of two key transcriptional regulators of adipogenesis (*PPARc2* and *C/EBPa*) (Olson and Soriano, 2011; Kalds et al., 2021). In the present study we found that *PDGFD* gene was strongly selected by Bamei Mutton sheep, combined with previous researches, indicating that *PDGFD* was involved in regulating the fat deposition process of sheep tail, but how to regulate this process still needs more in-depth research.

*GLIS1* is a zinc finger protein that acts as both an activator and repressor of transcription (Kim et al., 2002). The temporal and spatial expression of *GLIS1* is consistent with mesoderm differentiation during mouse embryonic development (Nakashima et al., 2002). Later, Tosic *et al.* discovered that *GLIS1*was highly expressed in bipotent muscle satellite cells. But when overexpressed, increased occupancy of *GLIS1* is observed at the promoters of adipogenic genes *Adipoq*, *Cebpa* and *Ucp1*, and drives brown adipogenesis both *in vitro* and *in vivo*, indicating that *GLIS1* was a novel pro-adipogenic transcription factor (Tosic et al., 2018). Most recently, *GLIS1*was detected as a candidate gene of selective signature of sheep tail phenotype (Luo et al., 2021). A non-synonymous point mutation (g.27807636G > T) was found within *GLIS1* in two fat-tailed Chinese indigenous sheep breeds (Mongolian sheep and Small Tail Han sheep) compared with two thin-tailed dairy sheep (DairyMeade and East Friesian), and resulted in a Pro to Thr substitution (Luo et al., 2021). In our study, *GLIS1* was also strongly selected in Bamei Mutton sheep compared with Mongolian sheep. Taken together, *GLIS1*, as a pro-adipogenic factor, may plays a key role in mesodermal cell differentiation during fetal development in fat-tailed sheep to initiate differentiation of pre-adipocytes and fat accumulation (Luo et al., 2021).

Nuclear receptor interacting protein one gene (*NRIP1*, also known as *RIP140*), encodes a nuclear protein also known as receptor-interacting protein 140 (RIP140). *RIP140* is widely expressed and plays an important role in regulating lipid and glucose metabolism (Leonardsson et al., 2004; Ho et al., 2011; Hochberg et al., 2015). *RIP140* interacts with multiple adipocyte-specific genes, such as *uncoupling protein 1* (*UCP1*), mitochondrial fatty acid transporter *carnitine palmitoyl transferase 1* (*CPT1*) and lipid droplet protein *cell death-inducing DFFA-like effector A* (CIDEA). The expression of these genes is characteristic of brown adipose tissue (Nautiyal et al., 2013). Previous studies in adipocyte cell models also revealed that *RIP140* functions as a corepressor of catabolic pathways, including fatty acid oxidation, oxidative phosphorylation, glycolysis and tricarboxylic acid cycle (Christian et al., 2005; Powelka et al., 2006). Moreover, Xu *et al.* conducted a genome-wide association study using phenotypes and genotypes of two breeds of contrasting tail types (Small-tailed and Large-tailed Han sheep breeds) to identify functional genes and variants associated with fat deposition, and revealed that *RIP140* was a strong candidate for fat deposition in the tails of sheep (Xu et al., 2017), which is consistent with our results.

It has been proved that androgen receptor (*Ar*) gene gets participate in lipid binding (Huang et al., 2009), and has a negative function in fat deposition in both mice and human beings (Rubinow et al., 2015; Kim et al., 2019). Adipose tissue macrophages express the androgen receptor (AR) and regulate adipose tissue remodeling. Thus, testosterone signaling in macrophages could alter the paracrine function of these cells and thereby contribute to the metabolic effects of androgens in men (Rubinow et al., 2015). In order to determine whether the loss of AR signaling in hematopoietic cells results in greater fat accumulation, Rubinow *et al.* performed a metabolic phenotyping study in male mice. C57BL/6J male mice (ages 12–14 weeks) underwent bone marrow transplant from either wild-type (WT) or *AR* knockout (ARKO) donors (n = 11–13 per group). Mice were fed a high-fat diet (60% fat) for 16 weeks. At baseline, 8 and 16 weeks, glucose and insulin tolerance tests were performed, and body composition was analyzed with fat-water imaging by MRI. No differences in body weight were observed between mice transplanted with WT bone marrow [WT (WTbm)] or ARKO bone marrow [WT (ARKObm)] prior to initiation of the high-fat diet. After 8 weeks of high-fat feeding, WT (ARKObm) mice exhibited significantly more visceral and total fat mass than WT (WTbm) animals. Resultant data indicate that AR signaling in hematopoietic cells influences body fat distribution in male mice, and the absence of hematopoietic AR plays a permissive role in visceral fat accumulation. These findings demonstrate a metabolic role for AR signaling in marrow-derived cells and suggest a novel mechanism by which androgen deficiency in men might promote increased adiposity (Rubinow et al., 2015). Kim *et al.* also discovered that blocking *AR* can decreases the expression of CPTI (one of long-chain fatty acid (LCFA) transport proteins) in the skeletal muscle, which reduces fat metabolism. Thus, reducing sex hormones or suppressing the sensitivity of AR can inhibit energy efficiency and fat metabolism by suppressing CPTI (Kim et al., 2019). However, the effect of *AR* gene on fat deposition in sheep is rarely reported. In our study, *AR* was identified as a promising candidate gene of sheep tail phenotype, and the same results were found in two Iranian thin- and fat-tailed sheep Breeds (Moradi et al., 2022). Combined with the above studies, we speculate that *AR* may promote energy efficiency and fat metabolism of sheep, thereby inhibit fat deposition in tail.

Fibroblast growth factor 9 (*FGF9*) is a protein-coding gene that plays an important role in the regulation of embryonic development, cell proliferation, cell differentiation, and cell migration (Moioli et al., 2015). *FGF9* expressed not only in white adipose tissue (WAT) of human, but also in brown adipose tissue (BAT) while exposed to cold, regulating the development of adipose tissue (Mejhert et al., 2010; Grefhorst et al., 2015). In mammals, WAT stores fat and BAT dissipates fat to produce heat. *FGF9* was selected as a candidate gene associated with tail phenotype in both our and a recent study (Moioli et al., 2015), indicating that *FGF9* may plays a role in fat deposition and metabolism of sheep, but the mechanism underlying it is unclear.

In addition to fat deposition, the number of caudal vertebrae also affects tail phenotype by affecting the length of sheep tail. It has been reported that *Vertnin* (*VRTN*) gene is a key candidate gene associated with the variation of vertebral number in sheep and pigs (Duan et al., 2018; Zhang et al., 2017). As a transcriptional inhibitor, *VRTN* independently regulates the expression of *BMP2* gene in the dorsoventral axis through combination with its regulatory sequence, thereby enabling normal development of embryos along the dorsoventral axis (Shao et al., 2017). In a study of genome-wide scan of selection signatures of sheep tail phenotype, Mastrangelo *et al.* identified *VRTN* as a key candidate gene; and the tail of the five fat-tailed sheep breeds where this signature was detected was definitively longer than the tail of the thin-tailed breeds (Mastrangelo et al., 2019). The same results were also found in Moioli *et al.*, Zhu *et al.*, and our study. In the current study, the tail length of Bamei Mutton sheep is longer than that of Mongolian sheep, with the caudal vertebrae number is 20–30 and 10–14, respectively. These results indicated that *VRTN* may be an important gene involved in regulating the development of sheep caudal vertebrae, and affect the tail phenotype of sheep by affecting the tail length.

Except for above mentioned genes, the rest of the candidate genes may also play important roles in regulating sheep tail phenotype, although there was not enough evidence at presence based on publicly available information. Therefore, further studies and experiments are needed to confirm the soles and mechanisms of these genes in formation of sheep tail phenotype. In conclusion, our results provide a strong foundation for studying the regulation of tail phenotype in sheep and do offer hope that the causal mutations and the mode of inheritance of this trait will soon be discovered by further experimentation.

## Data Availability

The data presented in the study are deposited in the SRA repository, accession number PRJNA1192386 (https://www.ncbi.nlm.nih.gov/bioproject/PRJNA1192386).
